# A Compend of Pharmacy

**Published:** 1887-03

**Authors:** 


A COMPEND OF PHARMACY. *By* F. E. STEWART, M.D., Ph.G. *Pp. 196. Philadelphia:* P. BLAKISTON, SON & COMPANY. *1886.*

This little book is No. 11 of the "Quiz-Compend" series. It is based on the well-known "Text Book of Pharmacy," by

Joseph P. Remington, and answers fully the purpose for which it is intended.

The author expressly states that it is not to be used as a text-book, but as an aid in the study of a larger work.

Employed in this way it will doubtless render assistance to the student of pharmacy.

